# A Comparative Analysis of Performance Simulation for PUI Detectors Based on Traditional Probability Model and the Vasyliunas and Siscoe Model

**DOI:** 10.3390/s24196233

**Published:** 2024-09-26

**Authors:** Yu Cao, Yuzhu Zhang, Xiaodong Peng, Changbin Xue, Bin Su

**Affiliations:** 1 Key Laboratory of Electronics and Information Technology for Space System, National Space Science Center, Chinese Academy of Sciences, Beijing 100190, China; caoyu22@mails.ucas.ac.cn (Y.C.);; 2 School of Computer Science and Technology, University of Chinese Academy of Sciences, Beijing 100049, China

**Keywords:** pickup ions detector, numerical simulation, Mass Resolution, geometric factor, count rates

## Abstract

In recent years, the enthusiasm for deep space missions has remained unabated, resulting in continuous advancements in the research field of space environment and particles. Many instruments carried on these missions have conducted detection of pickup ions (PUIs) in the solar system. For those instruments, simulation is an effective means and a crucial step for their performance optimization and future operation in-orbit. It holds great significance for the instrument’s in-orbit performance assessment, science operation optimization, and detection efficiency enhancement. In this paper, the traditional probability model and the Vasyliunas and Siscoe (V–S) model are used to generate the PUIs, which are the input for the simulation of the PUI detector. For further analysis, the numerical results of the simulation are processed to calculate the instrument’s geometric factor, mass resolution, and count rates. Then, two sets of experiments are carried out for the comparison of the traditional probability model and the V–S model. The results show that, for the simulation of the instrument in the design stage, the simulation results of the traditional probability model and the V–S model are not much different. However, for the simulation of the instrument performance in-orbit, the PUI data generated based on the V–S model gave a better result than those of the traditional probability model. This conclusion is of great significance for evaluating the detection ability of the PUI detector in future deep space explorations.

## 1. Introduction

Plasma is the fourth state of matter and widely exists in solar–terrestrial and interplanetary space. Human research on plasma originated in the 19th century and developed in the 20th century. By the middle of the 20th century, with the advancement of rocket and satellite, human beings officially opened the door to the detection of space plasma [1]. Comprehensive in situ detection is the main means to understand the outer heliospheric environment, especially the in-depth study of the interaction between PUIs and the solar wind [2].

Over the past half-century, numerous deep space missions have been equipped with the capability to detect PUIs. From the early missions, such as Pioneer 10/11 [3], Voyager 1/2 [4], AMPTE [5], Ulysses [6], Galileo [7], SOHO [8], and ACE [9], to the more recent missions, including Cassini [10], New Horizons [11], STEREO [12], Chang’E-1 [13], MAVEN [14], Rosetta [15], and Juno [16], the instruments onboard have conducted various degrees of PUI detection in the solar system. For the detection of low-density PUIs in the outer heliosphere, instruments require a large geometric factor and high mass resolution capabilities.

For the detection of low-temperature, low-speed, and low-density PUIs in the outer heliosphere, scholars have designed instruments that aim to achieve a low-energy range, high-energy resolution, and high-mass resolution detection [17]. Simulating the instrument is an effective means and a crucial step in optimizing its performance and preparing for future in-orbit operations. It holds significant importance in evaluating the instrument’s in-orbit performance and enhancing detection efficiency [18]. Regarding the existing simulation methods for these instruments, the simulation data of the PUI are typically modeled using traditional probability models [19,20]. These simulations are performed using commercial simulation software, where PUIs are specified by pitch angle range and energy range. Ultimately, the coordinates, angles, energies, and other information of ions that pass through the Retarding Potential Analyzer (RPA), Electrostatic Analyzer (ESA), and time-of-flight system (TOF) and reach a designated location are recorded. Through the statistical analysis of the recorded information, key indicators, such as energy resolution, pitch angle resolution, geometric factor, and mass resolution, can be obtained. Here, the geometric factor reflects the detection sensitivity of the instrument [21].

However, the aforementioned traditional probability models used to generate simulation data for PUIs are mathematical models that lack physical meaning. To a certain extent, they cannot objectively reflect the true conditions of the PUIs and, thereby, may affect the analysis of the instrument’s in-orbit performance. An early model of PUI velocity distributions was provided by V–S in 1976 [22], which describes PUIs that are generated by the interaction between interstellar neutral particles and the solar wind in the solar system. This model has been further refined in subsequent studies by scholars such as Chen [23,24] and McComas [25,26,27]. Therefore, in order to support the performance analysis of the PUI detector during the design phase and in-orbit, it is urgent to conduct research on simulation methods that are suitable for both conditions. To address this issue, this paper generates PUI simulation data based on the V–S model and processes the simulation results to obtain three performance indicators: geometric factor, mass resolution, and count rates. Two sets of experiments are carried out for the comparison of the traditional probability model and the V–S model.

The structure of this paper is as follows. In the next section, the basic detection principle and simulation process of the PUI detector are reviewed. In Section 3, a framework for the performance simulation and comparative analysis of the PUI detector is proposed. In Section 4, the traditional probability model and V–S model are tested, respectively, and the results are analyzed. The conclusions of this paper are summarized, and the outlook of future research is provided in Section 5.

## 2. Essential Preliminaries

### 2.1. The Basic Detection Principle of the PUI Detector

**Figure 1:**
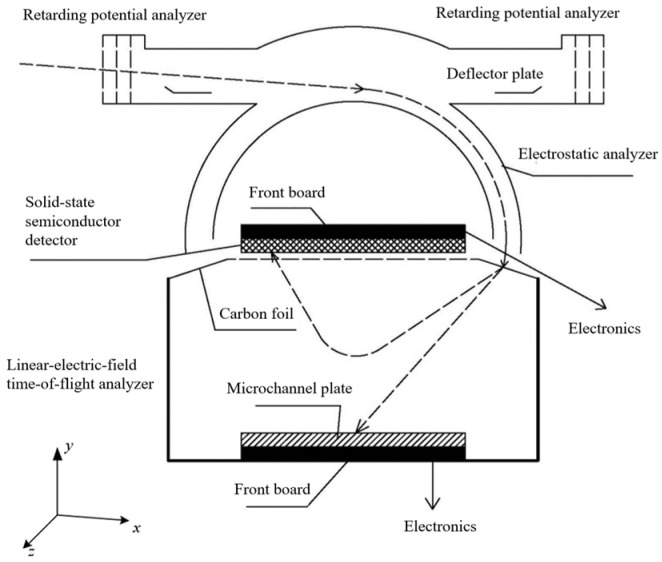
PUI detection principle and coordinate system.

The PUI detector is a space thermal plasma detection instrument and requires energy, directional and ion composition analysis. The basic detection principle of the instrument is illustrated in Figure 1, which primarily comprises an RPA, an ESA, and a linear field TOF system [17].

During operation, the RPA applies a positive voltage *V_RPA_*. For ions with mass *M*, charge *q*, and energy *E*, only those with energy *E* > *q*·*V_RPA_* can pass through the RPA and enter the ESA. The ESA consists of inner and outer concentric hemispherical plates, with the inner sphere biased negatively at voltage *V* and the outer sphere grounded. Ions are guided through the ESA slit by the radial electric field. The ions that can traverse the ESA must have an energy-to-charge ratio *E*/*q* satisfying *E*/*q* = *k*·*V*, where *k* is the ESA constant, which depends solely on the ESA geometry and is independent of the incident ion energy and voltage applied. After exiting the ESA, the ions are accelerated by a post-acceleration electric field and strike an ultrathin carbon foil, generating secondary electrons and an initial TOF pulse signal. The ions then undergo a single reflection flight within the linear field TOF system and ultimately impact the top solid-state semiconductor detector (SSD), generating a final TOF pulse signal, with their residual energy measured. By combining the TOF, residual energy, and *E*/*q*, the *M*, *q*, and *E* can be determined. The MCP is a high-gain multichannel electron multiplier, which has the function of charge amplification and has very high spatial and temporal resolution. In the PUI, the secondary electrons emitted from the carbon film hit the MCP to enhance the initial signal.

### 2.2. The Simulation Process of the PUI Detector

**Figure 2:**
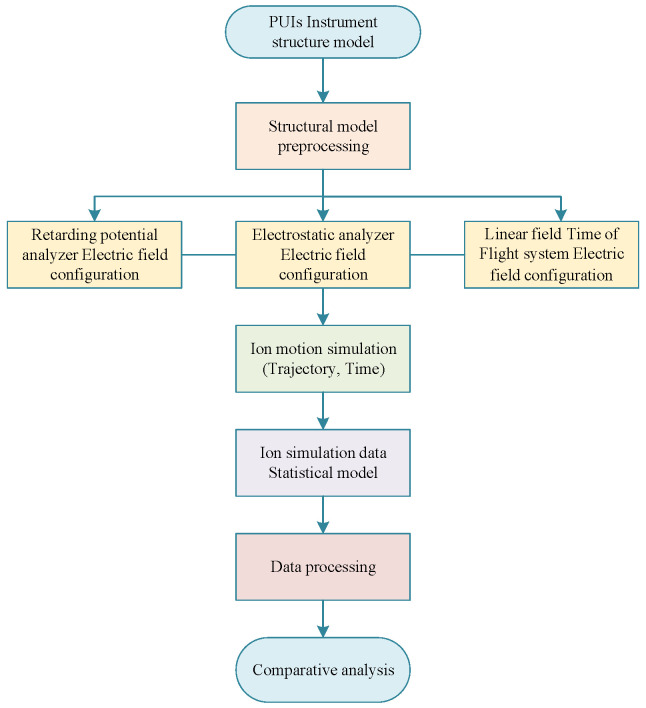
PUI detector simulation process.

Simulation is a crucial step in the instrument design process, involving an iterative optimization process with technical requirements. Through simulation, it can be determined whether the technical requirements are fulfilled while providing guidance for the design and optimization of the instrument. For PUI detectors, the simulation is conducted using the finite element simulation software SIMION 8.0 [28]. The simulation steps are shown in Figure 2.

Step 1: In the simulation process of the PUI detector, the first step is to construct a three-dimensional (3D) model of the detector using software such as Creo, COMSOL, AutoCAD, etc. This 3D model file is then converted into a .*stl* file format, which is further converted into a .*PA* file using SIMION software 8.0. Only the .*PA* file can be used for simulation in SIMION.

Step 2: The SIMION simulation software performs finite element meshing based on the established 3D detector structure and calculates the internal electric field of the detector by applying voltages to various electrodes, including those of the RPA, deflector plate, ESA, and TOF.

Step 3: The traditional input model of ions used in simulations is generated by the Monte Carlo method [29] based on statistical distributions. When simulating different performance parameters, the energy of incident ions is set as a single energy or established using Gaussian distribution. The position, direction, and other information are also established using a random uniform sampling method, generating a total of *N* ions. SIMION calculates the ions’ trajectories in the electric field based on the incident ions and the internal electric field of the instrument. Depending on the requirements, information such as the ion’s energy, azimuth, elevation, and time-of-flight at any target position can be statistically analyzed.

Step 4: Based on the statistical information of ion serial numbers, elevation angles, energies, positions, etc., the data information of these ions at the time of incidence can be traced. A data processing model is then constructed to calculate the performance indicators of the PUI detector.

## 3. Proposed Method

**Figure 3:**
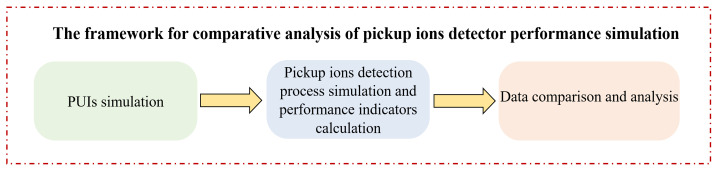
Framework for comparative analysis of the PUI detector performance simulation.

Based on the detection principles and procedures of the PUI detector described in Section 2, this study focuses on the analysis of the instrument’s in-orbit performance simulation. By altering the PUI detector’s input, the impact of different ion simulation models on its performance indicators and count rates is analyzed. Figure 3 illustrates the framework for the comparative analysis of the PUI detector performance simulation.

### 3.1. Mathematical Model of Ion Distribution

#### 3.1.1. Traditional Probability Models

The PUI simulation model used in traditional simulation during the design phase is established using the Monte Carlo method [30,31,32]. When simulating different performance indicators, the energy of incident ions is set as a single energy or modeled using a random uniform distribution or Gaussian distribution. Similarly, information such as position and direction is also modeled using a random uniform distribution or Gaussian distribution, generating a total of *N* ions as the incident source. The probability density function for the uniform distribution of the particle source is shown in Equation (1):

(1) $$p(x)=\left\{\begin{array}{ll} \frac{1}{b-a}, & a<x<b\\ 0, & else \end{array}\right.$$

where p(x) is the probability density function and *X* follows a uniform distribution over the interval [a, b], denoted as *X* ∼ *U* (a, b). Therefore, the uniform distribution model for the particle source is shown in Equation (2):

(2) $$F(x)=\left\{\begin{array}{ll} 0, & x<a\\ \frac{x-a}{b-a}, & a\leq x<b\\ 1, & x\geq b \end{array}\right..$$

The Gaussian distribution model for the particle source is as follows:

(3) $$p(x)=\frac{1}{\sqrt{2\pi }\sigma }\exp\left(\frac{{\left(x-\mu \right)}^{2}}{2\sigma^{2}}\right),-\infty <x<\infty ,$$

where p(x) is the probability density function and *X* is said to follow a Gaussian distribution with mean *μ* and variance $\sigma^{2}$, denoted as *X* ∼ *N*(*μ*, $\sigma^{2}$). The distribution function is given by the following:

(4) $$F(x)=\frac{1}{\sqrt{2\pi }\sigma }\int_{-\infty }^{t}\exp\left(\frac{{\left(t-\mu \right)}^{2}}{2\sigma^{2}}\right)dt$$

#### 3.1.2. Vasyliunas and Siscoe Model

Theoretically, the PUIs in the interstellar space can be predicted. For instance, the V–S model encompasses the ionization of incident neutral particles, the instantaneous isotropic distribution of scattering in the solar wind system, convection, and adiabatic cooling. In 2017, McComas adjusted the V–S model, and the representation of McComas’s model is given in Equation (5) [25]:

(5) $$f(r,w)=\frac{3}{8\pi }\frac{\beta_{0}r_{0}^{2}}{ru_{sw}v_{b}^{3}}w^{-3/2}n_{HTS}\times \exp\left(-\frac{\lambda }{r}\frac{\theta }{\sin\theta }w^{-3/2}\right)\Theta (1-w)$$

where r is the distance from the Sun, w is the ratio of the PUIs velocity v to the injection velocity $v_{b}$, $\beta_{0}$ represents the ionization rate normalized to *r*_0_ = 1 AU, $u_{sw}$ denotes the solar wind bulk velocity in the solar coordinate system, θ is the angle between the radial direction and the interstellar neutral hydrogen inflow direction, $n_{HTS}$ represents the density of interstellar neutral hydrogen at the bow shock at the upwind end, λ represents the size of the interstellar neutral hydrogen ionization cavity, and Θ(x) represents the Heaviside step function, whose expression is given below:

(6) $$\Theta (x)=\left\{\begin{array}{l} 0,x<0\\ 0.5,x=0\\ 1,x>0 \end{array}\right..$$

### 3.2. Detector Performance Indicators

In the simulation process of ion instruments, geometric factor and mass resolution are two crucial performance indicators that directly show the detection capability and precision of the instrument. The calculation and optimization of these two indicators are paramount to enhancing the overall performance of mass spectral analysis. Consequently, computations have been conducted specifically targeting the geometric factor and mass resolution of the PUI detector. Based on the statistical information of ion parameters obtained in SIMION, the two indicators mentioned above can be derived. This section delves into the detailed calculation for these two performance indicators.

#### 3.2.1. Geometric Factor

The geometric factor describes the efficiency of a particle instrument in collecting and detecting ions. Specifically, it quantifies the probability of a particle traversing through various components of the detector (such as RPA, ESA, etc.) and ultimately reaching the solid-state semiconductor detector. The geometric factor is typically related to the design of the detector (such as aperture size), which determines the length and shape of the ion flight path. Enhancing the geometric factor means an improvement of the ion capture and detection capabilities of the instrument, thereby boosting overall sensitivity [33]. The detailed calculation method is given below.

During the simulation process, ions are emitted at the entrance located on the left end of the RPA. By tracing the serial numbers of ions that successfully pass through the ESA to their positions at the time of incidence, statistical data can be filtered to obtain the pitch angle *β*, energy *E*, and position coordinates (*X*, *Y*, *Z*) of the ions at the time of incidence that can traverse the ESA. Based on this information, the *E*-*β* phase space distribution diagram of the ion beam and the count distribution diagram of ions in the geometric detection area can be plotted within the left-view *y*-*z* plane (coordinate definition, see Figure 1). Since the ESA is a rotational symmetry structure, the geometric factor can be calculated as follows:

(7) $$G=\alpha \int \int \int dA_{act}\cdot \frac{dE}{E}\cdot d\beta ,$$

where α, *β*, *E*, and $A_{act}$ represent the azimuth angle, pitch angle, energy, and active areas, respectively. The active areas are the product of the geometric detection area and the detection efficiency. The detection efficiency is the ratio of the number of ions within the detection area to the total number of emitted ions:

(8) $$A_{act}=S_{\mathrm{detector}}\cdot \eta_{\mathrm{detector}},$$

(9) $$\eta_{\mathrm{detector}}=\frac{n_{S}}{N},$$

where $S_{detector}$ represents the geometric detection area, $\eta_{detector}$ denotes the detection efficiency, $n_{s}$ is the number of ions within the detection area, and *N* is the total number of emitted ions. Given that the simulation results in this paper are discrete data, the discrete formula for the geometric factor is employed for the calculation, as shown in Equation (10) [12]:

(10) $$G\approx \alpha \sum_{i}\sum_{j}\Delta \theta_{i}\left(\Delta E_{j}/E_{j}\right){\left(A_{act}\right)}_{ij},$$

where $\Delta \theta_{i}\;$ is the elevation angle step size for step *i*, $E_{j}$ and $\Delta E_{j}$ are the energy and energy step size for step *j*, and ${\left(A_{act}\right)}_{ij}$ is the active areas of steps *i* and *j*.

#### 3.2.2. Mass Resolution

Mass resolution refers to the ability of a particle detector to distinguish ions with similar masses. High resolution implies the capability to differentiate between two ions with very close masses, which is crucial for the exploration of PUIs in complex space environments. Below is a detailed introduction to the simulation method for calculating the mass resolution.

During the simulation process, the SRIM (Stopping and Range of Ions in Matter) [34] simulation software is first utilized to simulate the trajectories and exit characteristics of ions using the carbon foil, with statistics collected on the energy loss and scattering of ions after exiting the carbon foil. Then, in SIMION, using the ions that went through the carbon foil as the input, ions are emitted at the carbon foil, and at the SSD, the serial numbers, time of flight, pitch angles, energies, positions, and other information of the ions are recorded and statistically analyzed.

The time-of-flight dataset {T} of the ions is then subjected to Gaussian fitting, resulting in the following Gaussian function:

(11) $$f_{T}(T)=\tau \exp\left\{\frac{-{\left(T-\mu_{T}\right)}^{2}}{2\sigma_{T}^{2}}\right\},$$

where $\mu_{T}$ represents the mean, which is the central value of the time-of-flight spectrum; τ is the peak of the Gaussian curve; and $\sigma_{T}$ is the standard deviation of the Gaussian curve, which corresponds to the peak width. The broadening of the time-of-flight spectrum is calculated based on the following model:

(12) $$f_{T}(T^{*})-\frac{\tau }{2}=0.$$

The zeros of the above model are solved as $x_{left}^{*}\in \left(\min\left(T\right),\mu_{T}\right),\;x_{right}^{*}\in \left(\mu_{T},\max\left(T\right)\right)$, and the broadening of the Gaussian-fitted time-of-flight spectrum is calculated as follows:

(13) $$FWHM=\Delta \tau =x_{right}^{*}-x_{left}^{*}.$$

The mass resolution $MSR=M/\Delta M=(\mu_{T}/\Delta \tau )/2$ can be derived from the ratio of the peak value to the broadening of the time-of-flight spectrum, where Δτ is the full width at half maximum (FWHM).

### 3.3. Calculation Method of Count Rates

Count rates refer to the number of particles recorded by a detector within a given time interval, commonly measured in counts per second (*cps*) or counts per minute (*cpm*). Count rates are a vital parameter in particle detection, which can be influenced by various factors such as the detector’s sensitivity, the particle’s energy, species, and incident angle. Additionally, solar activity, geomagnetic activity, and the orbital position of the satellite can also affect count rates. As a crucial parameter in particle physics, count rates are of significant importance for space environment monitoring, radiation monitoring, and other related fields. The calculation and simulation of these three indicators are essential for predicting the overall performance of PUI detectors [35,36].

The count rates can be calculated using Equation (14) (e.g., Nicolaou et al. 2014 [37]):

(14) $$c=\frac{2}{m^{2}}G^{E}E^{2}f(v)=\frac{1}{2}G^{E}v^{4}f(v),$$

where *c* represents the count rates, $G^{E}$ denotes the geometric factor of the detector, *E* is the energy of the PUIs, and f(v) represents the velocity distribution function of the PUIs. When the distribution of PUIs follows the Gaussian distribution model, the expression for the velocity distribution of PUIs can be obtained as follows:

(15) $$f(v)=\frac{1}{\sqrt{2\pi }\sigma }\exp\left(\frac{{\left(v-\mu \right)}^{2}}{2\sigma^{2}}\right),-\infty <v<\infty .$$

When the distribution of PUIs follows the V–S model, the function f(r,w) can be transformed into a function of f(v), with the specific transformation detailed as follows:

(16) $$f(v)=f(r,w)=\frac{3}{8\pi }\frac{\beta_{0}r_{0}^{2}}{ru_{sw}v_{b}^{3}}{\left(\frac{v}{v_{b}}\right)}^{-3/2}n_{HTS}\times \exp\left(-\frac{\lambda }{r}\frac{\theta }{\sin\theta }{\left(\frac{v}{v_{b}}\right)}^{-3/2}\right)\Theta \left(1-\frac{v}{v_{b}}\right).$$

The conversion between energy and velocity is achieved through the following function:

(17) $$E=\frac{1}{2}mv^{2},$$

where *E* represents the energy of the PUIs, *v* denotes the velocity of the PUIs, and *m* is the mass of a specific specie in the PUIs.

## 4. Experiment and Results

Based on the above methodology, experiments were conducted using software such as SIMION, SRIM, and Creo within the overall framework of the detector performance indicator calculation method. These experiments encompassed both traditional probability models and the V–S model, and their results were compared with real orbital data. The detailed elaboration is as follows.

Table 1 presents the steps for the experiments. Below is the detailed description of the calculation results of various performance indicators proposed for the PUI detector.

### 4.1. Comparison of Simulation Results between Traditional Models and the V–S Model

In this subsection, when simulating the aforementioned performance indicators, the energy of incident ions is modeled using either a single energy, a random uniform distribution, or a Gaussian distribution model. The position, direction, and other relevant information are modeled using either a random uniform distribution or a Gaussian distribution model. For example, the energy is set to obey the uniform distribution on the interval (830, 1200), and the pitch angle is set to obey the uniform distribution on the interval (−11, 0) during the calculation of the geometric factors using the traditional model. During the simulation process, particles are emitted at *X* = 10 mm, and their serial numbers, pitch angles, energies, positions, and other information are recorded at *Y* = 426 mm (after passing through the ESA). By tracing the serial numbers of particles that have passed through the ESA, the energies, pitch angles, and other information of these particles at the time of incidence can be retrieved.

#### 4.1.1. Geometric Factor Comparison

(1) Results of traditional probability models

The specific simulation parameters for the distribution of traditional probability models are shown in Table 2.

The figure below shows the simulation results of the response distribution in the *E*-*β* space of the instrument under the simulation of the traditional probability model.

**Figure 4:**
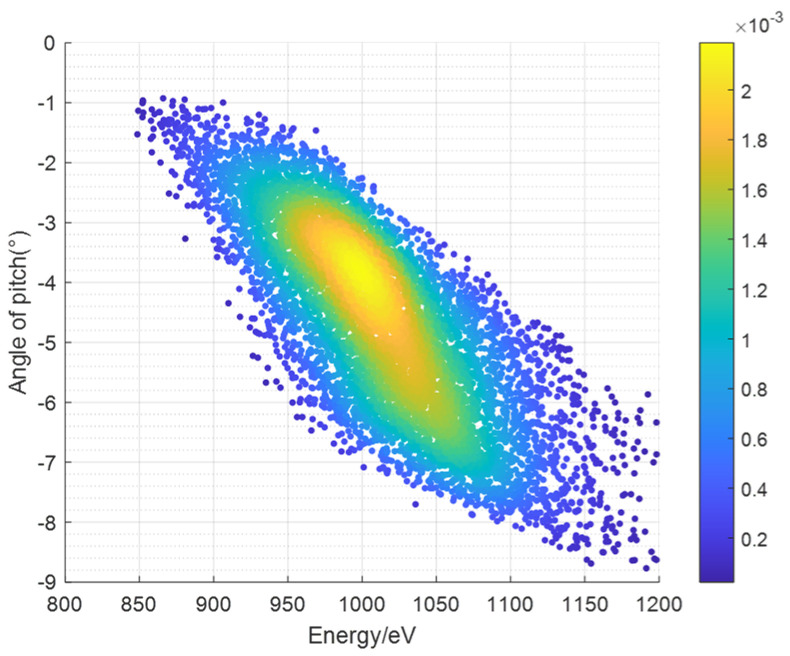
*E*-*β* phase space distribution diagram of ion beam simulated by the traditional probability model.

Taking the azimuthal angle field of view *α* = 15° for a single channel and summing over the E-β phase space shown in Figure 4 using Equation (10), the geometric factor of a single azimuthal angle channel is obtained, which is 2.788 × 10^−1^ cm^2^·sr·eV/eV.

(2) Results of V–S model

In McComas’ study [25], it showed that, at 30 AU and 90 AU, the PUI densities were 4.2 × 10^−4^/cm^3^ and 2.2 × 10^−4^/cm^3^, respectively. Based on the power law extrapolation model presented in the article,

(18) $$\rho_{PUI}=Ar^{\gamma },$$

where $\rho_{PUI}$ represents the PUI density, A is the amplitude coefficient, r is the distance from the Sun, and γ represents the radial dependencies coefficient, which is given as −0.58 in the article. Based on the PUI densities at 30 AU and 90 AU, the amplitude coefficient A is calculated to be 0.003. Therefore, using the power-law extrapolation method, the PUI density at 38 AU is calculated as follows:

(19) $$\rho_{PUI}=0.003\times 38^{-0.58}=3.66\times 10^{-4}/{\mathrm{cm}}^{3}.$$

Based on the parameters of the PUI detector, the simulated incident particle count is calculated using the following formula:

(20) $$N=SVt\rho_{PUI},$$

where N represents the simulated incident particle count, S is the cross-sectional area of the instrument facing the direction of the detector’s movement, V is the satellite’s velocity, t is the detection time, and $\rho_{PUI}$ is the PUI density. The final calculation shows that the simulated incident particle count within one day should be 1,554,876. When the orbit r is 38 AU, according to the V–S model f(r,w) and the relationship between energy and velocity (Equation (17)), the energy can be fitted to a Gaussian distribution with a mean of 942.2620 and a variance of 99.6550, and the interval (842.0325, 1069.171) is within the range of 3 sigma. Therefore, to maintain consistency with the variables of the 4.2 experiment, in this experiment, we set the energy range to U (842.0325, 1069.171). The initial parameters for the V–S model distribution simulation process are shown in Table 3 (In order to maintain consistency with subsequent experiments, this energy range is consistent with the energy band of SWAP).

**Figure 5:**
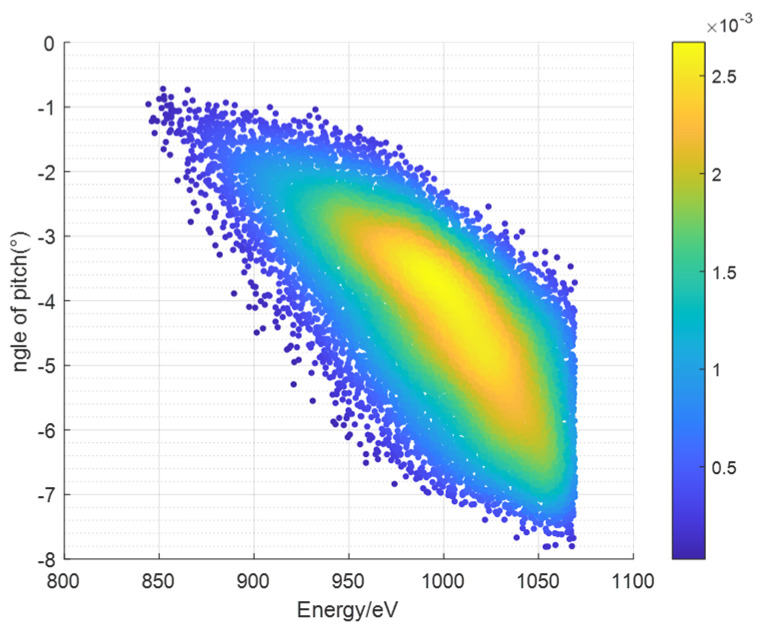
*E*-*β* phase space distribution diagram of ion beam simulated by the V–S model.

Figure 5 shows the simulation results of the response distribution in the *E*-*β* space of the instrument under the simulation of the V–S model distribution.

Taking the azimuthal angle field of view *α* = 15° for a single channel and summing over the *E*-*β* phase space shown in Figure 5 using Equation (10), the geometric factor for one azimuthal angle channel is obtained, which is 2.876 × 10^−1^ cm²·sr·eV/eV. Comparing this result with that obtained from the traditional probability model calculations, the difference is found to be 3.16%.

#### 4.1.2. Mass Resolution Comparison

(1) Results of traditional probability models

**Figure 6:**
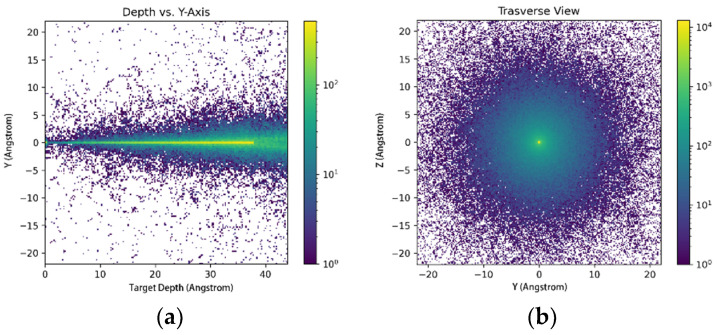
Trajectory simulation of ions in the carbon foil: (**a**) transverse view; (**b**) depth vs. y-axis.

The mass resolution of the instrument is determined by the broadening of the time-of-flight spectrum measured by the TOF. The time-of-flight broadening is primarily caused by the energy broadening of ions exiting the ESA and the energy and direction broadening induced by ions passing through the carbon foil. Ions of different compositions exhibit different responses when passing through the carbon foil. The simulations are conducted separately for H^+^, He^+^, He^2+^, C^+^, N^+^, O^+^, and Ne^+^ components. In the simulation process, the SRIM simulation software is first utilized to simulate the trajectories and exit characteristics of ions (including acceleration at −15 kV) within the carbon foil, as shown in Figure 6. The distribution of the ion incident source conforms to the energy broadening characteristics of ions exiting the ESA. The energy loss (ΔE) of ions after exiting the carbon foil is statistically determined to be 520 eV.

**Figure 7:**
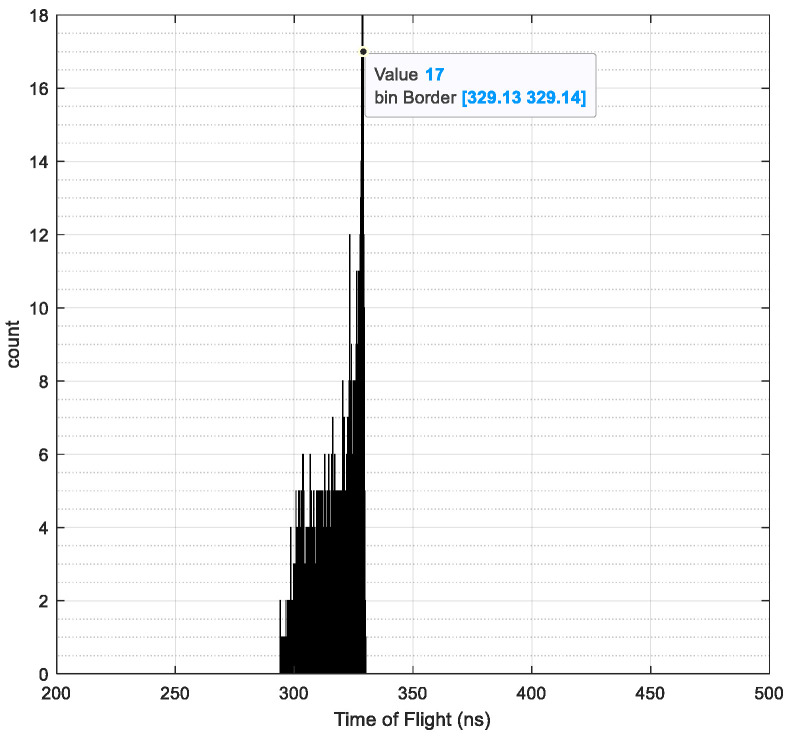
Traditional probability model for H^+^ time-of-flight spectrum.

Taking the ions exiting the carbon foil as the input source and using the simulation parameters set in Table 2, the following simulation is performed by SIMION. Taking H^+^ ions with an initial energy range from *U* (830, 1200) eV, charge *q* = 1, and mass *M* = 1 as an example, the time-of-flight spectrum is illustrated in Figure 7. Regarding the simulation of flight spectrum, previous research has been conducted by scholars such as Mobius [5] and Galvin [12], who adopted a time-of-flight magnitude on the order of nanoseconds (ns). Therefore, this paper also adopts the nanosecond scale.

The full width at half maximum (FWHM) is calculated to be 3.02, and the time of flight is 329.13. Using the peak center and broadening ratio of the time-of-flight spectrum in conjunction with Equations (12) and (13), the mass resolution M/ΔM can be obtained as $\mu_{T}$/Δτ/2 = 54.4901. Similarly, for a 4.4 nm carbon foil, the simulated time-of-flight results for ion species ranging from H^+^ to Ne^+^ are presented in Table 4.

(2) Results of V–S model

**Figure 8:**
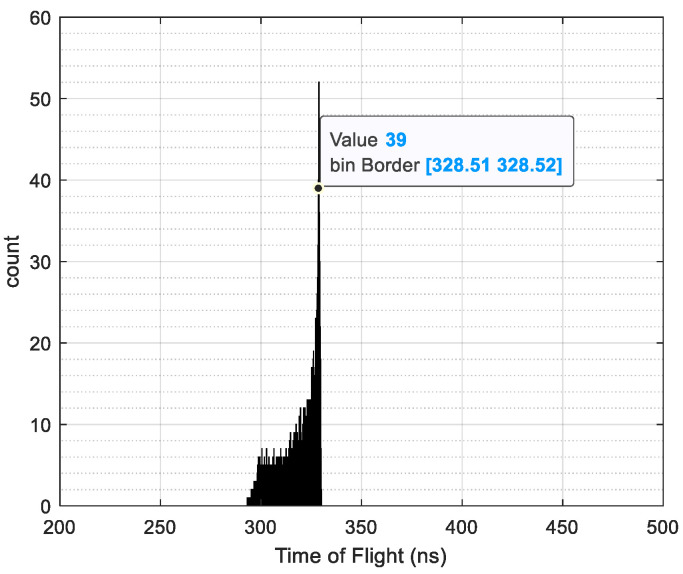
V–S model for H^+^ time-of-flight spectrum.

Based on the orbital parameters of the New Horizons spacecraft (abbreviated as New Horizons), the PUI detector is designed to cover an energy range of 0.023 to 7.87 keVq^−1^ utilizing 64 sampling points distributed logarithmically across equal channel widths. The specific simulation parameters are set as detailed in Table 3. Employing SIMION 8.0 software, simulations were conducted using carbon foil-emitted ions as the input source. Taking H+ ions with an initial energy range from U (842.0325, 1069.171) eV, charge *q* = 1, and mass *M* = 1 as an example, the resulting time-of-flight spectrum is illustrated in Figure 8.

The FWHM is 3, and the time of flight is 328.51. By combining the peak center and broadening ratio of the time-of-flight spectrum with Equations (12) and (13), the mass resolution M/ΔM can be obtained as M/ΔM = $\mu_{T}$/Δτ/2 = 54.3891. Comparing this result with those obtained using traditional probability model calculations reveals a difference of 0.19%. Similarly, for a 4.4 nm carbon foil, the simulated time-of-flight results for ion species H^+^ to Ne^+^ are presented in Table 5.

**Figure 9:**
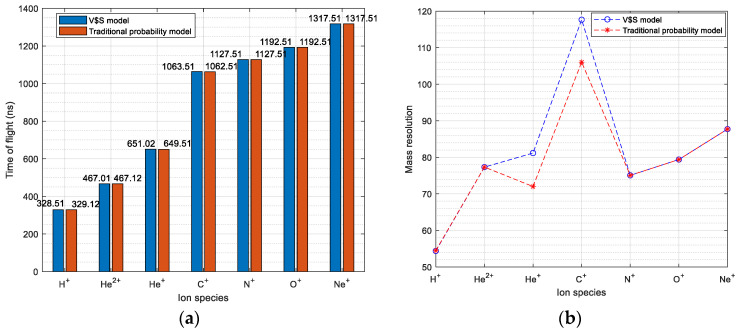
Comparison of simulation results between the V–S model and traditional probability model: (**a**) time of flight; (**b**) mass resolution.

The comparison between the experimental results of the V–S model and traditional probability distribution is shown in Figure 9. It shows that the difference of time of flight for each specie is no more than 0.23%, while the difference of mass resolution is no more than 12.73%.

In summary, the comparison results of the geometric factor and mass resolution of the PUI detector obtained from the experiments of the traditional probability model and the V–S model are shown in Table 6. By comparison, we can observe that the similarity of the simulation results of the PUI detector geometric factor and mass resolution under the traditional probability model and the V–S model is 96.84% and 99.81%, respectively.

### 4.2. Comparison of Simulation Results with SWAP PUI Data

Launched by the United States in January 2006, New Horizons is planned to explore Pluto and its largest moon Charon and asteroid populations located in the Kuiper Belt [11]. On 14 July 2015, the spacecraft flew by the Pluto system and entered within 11.5 Pluto radii (RP, where 1 RP = 1187 km). The SWAP instrument carried by New Horizons is designed to measure solar winds and PUIs.

Data related to interstellar PUIs of hydrogen measured by SWAP at approximately 58 AU from the Sun can be accessed from the Princeton Space Physics website. These measurements have revealed numerous distributions of interstellar hydrogen PUIs, their behavior within interplanetary shocks, and extrapolations of their characteristics at the heliospheric termination shock. The release of this data has improved and extended previous analyses of interstellar PUI measurements, extending them to ~58 AU, and represents a primary interstellar PUI dataset for the heliosphere. To ensure consistency in the orbit, based on the aforementioned theoretical models, we select data from an orbit at 38 AU [26] and use data at an energy of *E* = 870.6289 eV as the control sample dataset. The data can be found at [38].

According to the relationship between energy and velocity (Equation (17)), the velocity distribution under the traditional model is obtained. Table 7 presents the parameter values for each symbol of the velocity distribution in the traditional probability model (Equation (15)).

The calculated count rate under the traditional probability model is 3.4877 × 10^3^ ± 3.4877. Table 8 presents the parameter values for each symbol in the V–S model (Equation (16)).

The calculated count rate under the V–S model is 3.0763 × 10^3^ ± 14.3197. Similarly, the count rates at other energy points within the simulated energy range are calculated. Ultimately, the comparison of count rates between the traditional probability model, the V–S model, and the SWAP PUI data is shown in Table 9.

**Figure 10:**
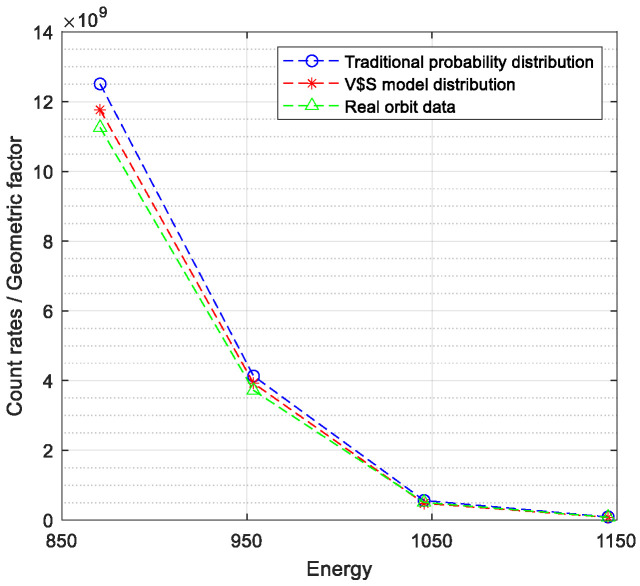
Figure of count rates/geometric factor comparison.

Due to the differences of the geometrical factors, in order to eliminate the influence of their difference, the ratio of the count rate to geometrical factor is compared for all three, which is shown in Figure 10.

According to Figure 10, the difference between the traditional probability model simulation results and the SWAP PUI data at each energy point is less than 11.1%, while the difference between the V–S model simulation results and the SWAP PUI data is less than 5.5%. Therefore, for the simulation of the detector in-orbit, the V–S model performs better than the traditional probability model. The V–S model simulates the distribution of PUI produced by the interaction of interstellar neutral particles and the solar wind in the solar system, which has more specific physical implications. Hence, in principle, the V–S model should be closer to the actual data, which is consistent with the result in our experiment.

## 5. Conclusions and Future Work

In this paper, a comparative analysis of the performance simulation of the PUI detector based on the traditional probability model and the V–S model is carried out. The design of the deep space exploration instrument is dynamically correlated with the performance analysis process. Based on the system engineering concept, method, and digital means, the ability of instrument performance evaluation and trend prediction is improved. By simulating the PUI detector based on the two ion input models, the traditional probability model and the V–S model, the numerical results of the simulation output are processed, and the PUI detector geometry factor, mass resolution, and count rates are calculated. Two sets of experimental results are compared. It can be observed that the difference of the simulation results of the PUI detector geometric factor and mass resolution under the traditional probability model and the V–S model is 3.16% and 0.19%, respectively, so it can be concluded that, for the simulation of the instrument in the design phase, the simulation results of the traditional probability model and the V–S model are not much different, and they can both support the performance simulation of the instrument in the design phase. In the comparison of simulation results with SWAP PUI data, the V–S model is closer to the results of the actual detection data, so it can be concluded that, for the simulation of the instrument in-orbit, the V–S model performs better than the traditional probability model. This conclusion is of great significance for evaluating the detection ability of the PUI detector and predicting the deep space detection. In the future, based on the method proposed in this paper, the performance indicators of particle detection instruments can be simulated by adopting a more accurate space plasma velocity distribution model so as to support their in-orbit performance evaluation.

## Figures and Tables

**Table 1:** Steps for experimental calculation and comparison process.

Experimental Calculation and Comparison Process
Begin Step 1: Select the V–S theoretical distribution model. Step 2: Input parameters such as *r*, *θ*, *λ*, etc. Step 3: Calculate parameters such as the number and energy of PUIs. Step 4: Generate .ion file data based on PUI parameters. Step 5: Input into SIMION for detector simulation. Step 6: Output information parameters such as particle number, pitch angle, energy, position, etc. Step 7: Perform data processing to calculate PUI detection performance indicators. Step 8: Calculate count rates. Step 9: Repeat the above steps by selecting traditional probability models. Step 10: Compare the output results of the two models with real orbital data. End

**Table 2:** Initial parameters for the simulation process of traditional probability models.

Parameter	Incident Ion Number	ESA Voltage (V)	RPA Voltage (V)	Energy Range (eV)	Pitch Angle Range (°)
Value	1,000,000	−235	0	*U* (830, 1200)	*U* (−11, 0)

**Table 3:** Initial parameters for the simulation process of the V–S models.

Parameter	Incident Ion Number	ESA Voltage (V)	RPA Voltage (V)	Energy Range (eV)	Pitch Angle Range (°)
Value	1,554,876	−235	0	*U* (842.0325, 1069.171)	*U* (−11, 0)

**Table 4:** Simulation results of time of flight for H^+^~Ne^+^ under the traditional probability model (carbon foil thickness is 4.4 nm).

Ion Type	Time of Flight (ns)	Time of Flight Half Height Width (ns)	Initial Energy *E*0/*q* (eV/e)	Energy *E*0 (eV)	Quantity of Electric Charge	Mass Number	Mass Resolution
H^+^	329.12	3.02	1013.4	1016.0588	1.0368	1.0997	54.4901
He^2+^	467.12	3.02	2027.2	2032.2832	2.0057	4.4481	77.3377
He^+^	649.51	4.51	1013.5	1016.1892	1.0029	4.3183	72.0078
C^+^	1062.51	5.01	1012.7	1015.7998	1.0033	11.5741	106.0389
N^+^	1127.51	7.51	1012.8	1015.8838	1.0033	13.0391	75.0672
O^+^	1192.51	7.51	1013.0	1016.3168	1.0036	14.6238	79.3948
Ne^+^	1317.51	7.51	1014.5	1017.5417	1.0033	17.7655	87.7170

**Table 5:** Simulation results of time of flight for H^+^~Ne^+^ under the V–S model (carbon foil thickness is 4.4 nm).

Ion Type	Time of Flight (ns)	Time of Flight Half Height Width (ns)	Initial Energy *E*0/*q* (eV/e)	Energy *E*0 (eV)	Quantity of Electric Charge	Mass Number	Mass Resolution
H^+^	328.51	3.02	993.4582	997.4629	1.0042	1.1119	54.3891
He^2+^	467.01	3.02	2004.2	2010.6922	2.0069	4.4739	77.3195
He^+^	651.02	4.01	994.2269	998.2393	1.0042	4.3640	81.1746
C^+^	1063.51	4.52	993.6562	997.9728	1.0046	11.6772	117.6449
N^+^	1127.51	7.51	994.1924	998.5700	1.0046	13.1567	75.0672
O^+^	1192.51	7.51	994.1826	998.6534	1.0047	14.7346	79.3948
Ne^+^	1317.51	7.51	994.7709	999.1730	1.0047	17.9262	87.7170

**Table 6:** Comparison of simulation results between the V–S model and traditional probability model: geometric factor and mass resolution.

Performance Indicators	Traditional Probability Model Results	V–S Model Results
Geometric Factor	2.788 × 10^−1^ cm^2^·sr·eV/eV	2.876 × 10^−1^ cm^2^·sr·eV/eV
Mass Resolution	≥54.4901	≥54.3891

**Table 7:** Table of parameters for each symbol in the traditional probability model.

Symbol	Value
μ	4.662×10^3^
σ	16.0686

**Table 8:** Table of parameters for each symbol in the V–S model.

Symbol	Value
r	10AU
$w=v/v_{b}$	v/440 km/s
$v_{b}=|v_{H}-u_{sw}|$	440 ± 1 km/s
$\beta_{0}$	(5.6 ± 0.6) × 10^−7^/s
$u_{sw}$	418 ± 1 km/s
θ	(252.2°, 9.0°)
$n_{H,TS}$	0.1268 ± 0.0040(λ) ± 0.0033(method) 0.0011(stat.) cm^−3^
λ	3.8 ± 0.4au
Θ(x)	0(x < 0), 1/2(x = 0), 1(x > 0)
$v_{H}$	22 km/s

**Table 9:** Table of count rate comparison.

Energy (eV)	Traditional Models	V–S Model	SWAP PUI Data
870.6289	3.4877 × 10^3^ ± 16.2347	3.0763 × 10^3^ ± 14.3197	2.6270 × 10^3^ ± 2.2931
953.5612	1.1520 × 10^3^ ± 4.4702	1.0258 × 10^3^ ± 3.9803	8.6772 × 10^2^ ± 1.3179
1045.7081	1.5684 × 10^2^ ± 0.5061	1.2521 × 10^2^ ± 0.4040	1.1814 × 10^2^ ± 0.4863
1145.2268	2.4993 × 10^1^ ± 0.0672	2.0424 × 10^1^ ± 0.0549	1.8825 × 10^1^ ± 0.1941

## Data Availability

The data presented in this study are available on request from the corresponding author.

## References

[1] Gao T.F. (2023). Study on the High-Resolution Detector for Outer Heliospheric Pickup Ions. Ph.D. Thesis.

[2] Schwadron N.A., McComas D.J. (2010). Pickup Ions from Energetic Neutral Atoms. Astrophys. J. Lett..

[3] Stilwell D.E., Joyce R.M., Teegarden B.J., Trainor J.H., Streeter G., Bernstein J. (2007). The Pioneer 10/11 and Helios A/B cosmic ray instruments. IEEE Trans. Nucl. Sci..

[4] Bridge H.S., Belcher J.W., Butler R.J., Lazarus A.J., Mavretic A.M., Sullivan J.D., Vasyliunas V.M. (1977). The plasma experiment on the 1977 voyager mission. Space Sci. Rev..

[5] Möbius E., Hovestadt D., Klecker B., Scholer M., Gloeckler G., Ipavich F.M. (1985). Direct observation of He^+^ pick-up ions of interstellar origin in the solar wind. Nature.

[6] Bame S.J., Mccomas D.J., Barraclough B.L., Phillips J.I., Sofaly K.J., Chavez J.C., Goldstein B.E., Sakurai R.K. (1992). The Ulysses solar wind plasma experiment. Astron. Astrophys. Suppl. Ser..

[7] Frank L.A., Ackerson K.L., Lee J.A., English M.R., Pickett G.L. (1992). The plasma instrumentation for the Galileo Mission. Space Sci. Rev..

[8] Hovestadt D., Hilchenbach M., Bürgi A., Klecker B., Laeverenz P., Scholer M., Neugebauer M. (1995). CELIAS-charge, element and isotope analysis system for SOHO. Sol. Phys..

[9] Gloeckler G., Cain J., Ipavich F.M., Tums E.O., Bedini P., Fisk L.A., Kallenbach R. (1998). Investigation of the composition of solar and interstellar matter using solar wind and pickup ion measurements with SWICS and SWIMS on the ACE spacecraft. Space Sci. Rev..

[10] Young D.T., Berthelier J.J., Blanc M., Burch J.L., Coates A.J., Goldstein R., Zinsmeyer C. (2004). Cassini Plasma Spectrometer Investigation. Space Sci. Rev..

[11] McComas D., Allegrini F., Bagenal F., Casey P., Delamere P., Demkee D., Weidner S. (2008). The Solar Wind Around Pluto (Swap) Instrument Aboard New Horizons. Space Sci. Rev..

[12] Galvin A.B., Kistler L.M., Popecki M.A., Farrugia C.J., Simunac K.D., Ellis L., Steinfeld D. (2008). The plasma and suprathermal ion composition (Plastic) investigation on the stereo observatories. Space Sci. Rev..

[13] Zhong J., Xie L., Zhang H., Li J.X., Pu Z.Y., Nowada M., Wang S.J. (2013). Chang’e-1 observations of pickup ions near the moon under different interplanetary magnetic field conditions. Planet. Space Sci..

[14] McFadden J.P., Kortmann O., Curtis D., Dalton G., Johnson G., Abiad R., Jakosky B. (2015). Maven suprathermal and thermal ion compostion (static) instrument. Space Sci. Rev..

[15] Goldstein R., Burch J.L., Mokashi P., Broiles T., Mandt K., Hanley J., Webster J.M. (2015). The rosetta ion and electron sensor (IES) measurement of the development of pickup ions from comet 67p/c huryumov-gerasimenko. Geophys. Res. Lett..

[16] McComas D.J., Alexander N., Allegrini F., Bagenal F., Beebe C., Clark G., White D. (2017). The Jovian Auroral Distributions Experiment (Jade) On The Juno Mission To Jupiter. Space Sci. Rev..

[17] Gao T.F., Kong L.G., Su B., Zhang A. (2021). Design and simulation of the detector for outer heliosphere pickup ions. J. Beijing Univ. Aeronaut. Astronaut..

[18] Rodríguez-Pacheco J., Wimmer-Schweingruber R.F., Mason G.M., Ho G.C., Sánchez-Prieto S., Prieto M., Zong Q. (2020). The Energetic Particle Detector: Energetic particle instrument suite for the Solar Orbiter mission. Astron. Astrophys..

[19] Yu D.J. (1999). Numerical simulation of a cylindrical electrostatic analyzer. Chin. J. Space Sci..

[20] Kong L., Zhang A., Wang S., Sun Y., Zheng X., Dong Y. (2012). Numerical Simulation Analysis of Space Plasma Detector Based on SIMION. Chin. Space Sci. Technol..

[21] Wuest M., Evans D.S., McFadden J.P. (2007). Calibration of Particle Instruments in Space Physics.

[22] Vasyliunas V.M., Siscoe G.L. (1976). On the flux and the energy spectrum of interstellar ions in the solar system. J. Geophys. Res..

[23] Chen J.H., Bochsler P., Möbius E., Gloeckler G. (2014). Possible modification of the cooling index of interstellar helium pickup ions by electron impact ionization in the inner heliosphere. J. Geophys. Res. Space Phys..

[24] Chen J.H., Schwadron N.A., Möbius E., Gorby M. (2015). Modeling interstellar pickup ion distributions in corotating interaction regions inside 1AU. J. Geophys. Res. Space Phys..

[25] McComas D.J., Zirnstein E.J., Bzowski M., Elliott H.A., Randol B., Schwadron N.A., Weaver H. (2017). Interstellar Pickup Ion Observations to 38 au. Astrophys. J. Suppl. Ser..

[26] McComas D.J., Swaczyna P., Szalay J.R., Zirnstein E.J., Rankin J.S., Elliott H.A., Weaver H. (2021). Interstellar Pickup Ion Observations Halfway to the Termination Shock. Astrophys. J. Suppl. Ser..

[27] McComas D.J., Shrestha B.L., Swaczyna P., Rankin J.S., Weidner S.E., Zirnstein E.J., Weaver H.A. (2022). First High-resolution Observations of Interstellar Pickup Ion Mediated Shocks in the Outer Heliosphere. Astrophys. J..

[28] Manura D.J., Dahl D.A. (2008). SIMION 8.0 User Manual.

[29] Michael K. (2010). Monte Carlo methods in statistical physics: Mathematical foundations and strategies. Commun. Nonlinear Sci. Numer. Simul..

[30] Bundaleski N., Rakocevic Z., Terzc I. (2002). Optical properties of the 127° cylindrical energy analyzer used in LEIS experiments. Nucl. Instrum. Methods Phys. Res. B.

[31] Cui S.H., Wei Z., Jian H., Li X.T., Chen Q.H., Guo Y.X., Wu Z.Z. (2023). High-efficient particle-in-cell/Monte Carlo model for complex solution domain and simulation of anode layer ion source. Acta Phys. Sin..

[32] Jiao Z.L., Hu S.L., Jiang L.X. (2019). General algorithm for calculating space environmental exposure flux based on Monte-Carlo ray tracing. Spacecr. Environ. Eng..

[33] Chase L.M. (1973). The geometrical factor of large aperture hemispherical electrostatic analyzers. Rev. Sci. Instrum..

[34] Ziegler J.F. (2004). SRIM-2003. Nucl. Instrum. Methods Phys. Res. Sect. B Beam Interact. Mater. At..

[35] Randol B.M., McComas D.J., Schwadron N.A. (2013). Interstellar Pick-Up Ions Observed between 11 and 22 AU by New Horizons. Astrophys. J. Suppl. Ser..

[36] Elliott H.A., McComas D.J., Valek P., Nicolaou G., Weidner S., Livadiotis G. (2016). The New Horizons Solar Wind Around Pluto (SWAP) Observations of the Solar Wind from 11-33 au. Astrophys. J. Suppl. Ser..

[37] Nicolaou G., McComas D.J., Bagenal F., Elliott H.A. (2014). Properties of plasma ions in the distant Jovian magnetosheath using Solar Wind Around Pluto data on New Horizons. J. Geophys. Res. Space Phys..

[38] SWAP Count Rates Data. https://spacephysics.princeton.edu/missions-instruments/swap/pui-data-2021.

